# Elaboration of the Environmental Stress Hypothesis–Results from a Population-Based 6-Year Follow-Up

**DOI:** 10.3389/fpsyg.2016.01904

**Published:** 2016-12-15

**Authors:** Matthias Wagner, Darko Jekauc, Annette Worth, Alexander Woll

**Affiliations:** ^1^Department of Sports Science, University of KonstanzKonstanz, Germany; ^2^Department of Sports Psychology, Humboldt University of BerlinBerlin, Germany; ^3^Institute of Physical Education and Sports, University of Education KarlsruheKarlsruhe, Germany; ^4^Institute of Sports and Sports Science, Karlsruhe Institute of TechnologyKarlsruhe, Germany

**Keywords:** gross motor coordination problems, poor motor skills, mental health problems, overweight and obesity, physical inactivity

## Abstract

The aim of this paper was to contribute to the elaboration of the Environmental Stress Hypothesis framework by testing eight hypotheses addressing the direct impact of gross motor coordination problems in elementary-school on selected physical, behavioral and psychosocial outcomes in adolescence. Results are based on a longitudinal sample of 940 participants who were (i) recruited as part of a population-based representative survey on health, physical fitness and physical activity in childhood and adolescence, (ii) assessed twice within 6 years, between the ages of 6 and 10 years old as well as between the ages of 12 and 16 years old (Response Rate: 55.9%) and (iii) classified as having gross motor coordination problems (*N* = 115) or having no gross motor coordination problems (*N* = 825) at baseline. Motor tests from the Körperkoordinationstest, measures of weight and height, a validated physical activity questionnaire as well as the Strength and Difficulties Questionnaire were conducted. Data were analyzed by use of binary logistic regressions. Results indicated that elementary-school children with gross motor coordination problems show a higher risk of persistent gross motor coordination problems (*OR* = 7.99, *p* < 0.001), avoiding organized physical activities (*OR* = 1.53, *p* < 0.05), an elevated body mass (*OR* = 1.78, *p* < 0.05), bonding with sedentary peers (*OR* = 1.84, *p* < 0.01) as well as emotional (*OR* = 1.73, *p* < 0.05) and conduct (*OR* = 1.79, *p* < 0.05) problems in adolescence in comparison to elementary-school children without gross motor coordination problems. However, elementary-school children with gross motor coordination problems did not show a significantly higher risk of peer problems (*OR* = 1.35, *p* = 0.164) or diminished prosocial behavior (*OR* = 1.90, *p* = 0.168) in adolescence, respectively in comparison to elementary-school children without gross motor coordination problems. This study is the first to provide population-based longitudinal data ranging from childhood to adolescence in the context of the Environmental Stress Hypothesis which can be considered a substantial methodological progress. In summary, gross motor coordination problems represent a serious issue for a healthy transition from childhood to adolescence which substantiates respective early movement interventions.

## Introduction

Recent research indicates that children with motor coordination problems often show reduced physical fitness (e.g., Schott et al., 2007) as well as an increased risk in becoming overweight or obese (e.g., Cairney et al., 2010a), which could be explained by a reduced participation in physical activity (e.g., Rivilis et al., 2011) especially concerning team sports (e.g., Poulsen et al., 2007). To the extent of being integrated in a group or a team, it is well known that children with motor coordination problems face a variety of difficulties concerning social interaction including lower sociometric peer-preference scores (e.g., Livesey et al., 2011) or peer-victimization (e.g., Campbell et al., 2012).

Besides the effects of motor coordination-related social interaction problems on the children's family system and especially their parents (Stephenson and Chesson, 2008) who were—similar to the teachers—partially found to react in a negative way on comorbid behaviors such as inattention and task avoidance (Missiuna et al., 2006) but also to more frequently assist and encourage their children (Pless et al., 2001), a decreased participation in social activities (e.g., Sylvestre et al., 2013) first and foremost affects the child itself. In this regard, most frequently reported psychosocial outcomes of having motor coordination problems are a reduced self-worth (e.g., Skinner and Piek, 2001) which could likely develop due to bullying-experiences (Piek et al., 2005) and result in further decreased participation levels (Cairney et al., 2005b), less enjoyment of physical education classes, (Cairney et al., 2007), reduced perceived (Schoemaker and Kalverboer, 1994) or actual (e.g., Cummins et al., 2005) social competence skills, lower levels of perceived social support (e.g., Skinner and Piek, 2001), loneliness (e.g., Poulsen et al., 2007) as well as anxiety and depression (e.g., Missiuna et al., 2014).

**Figure 1:**
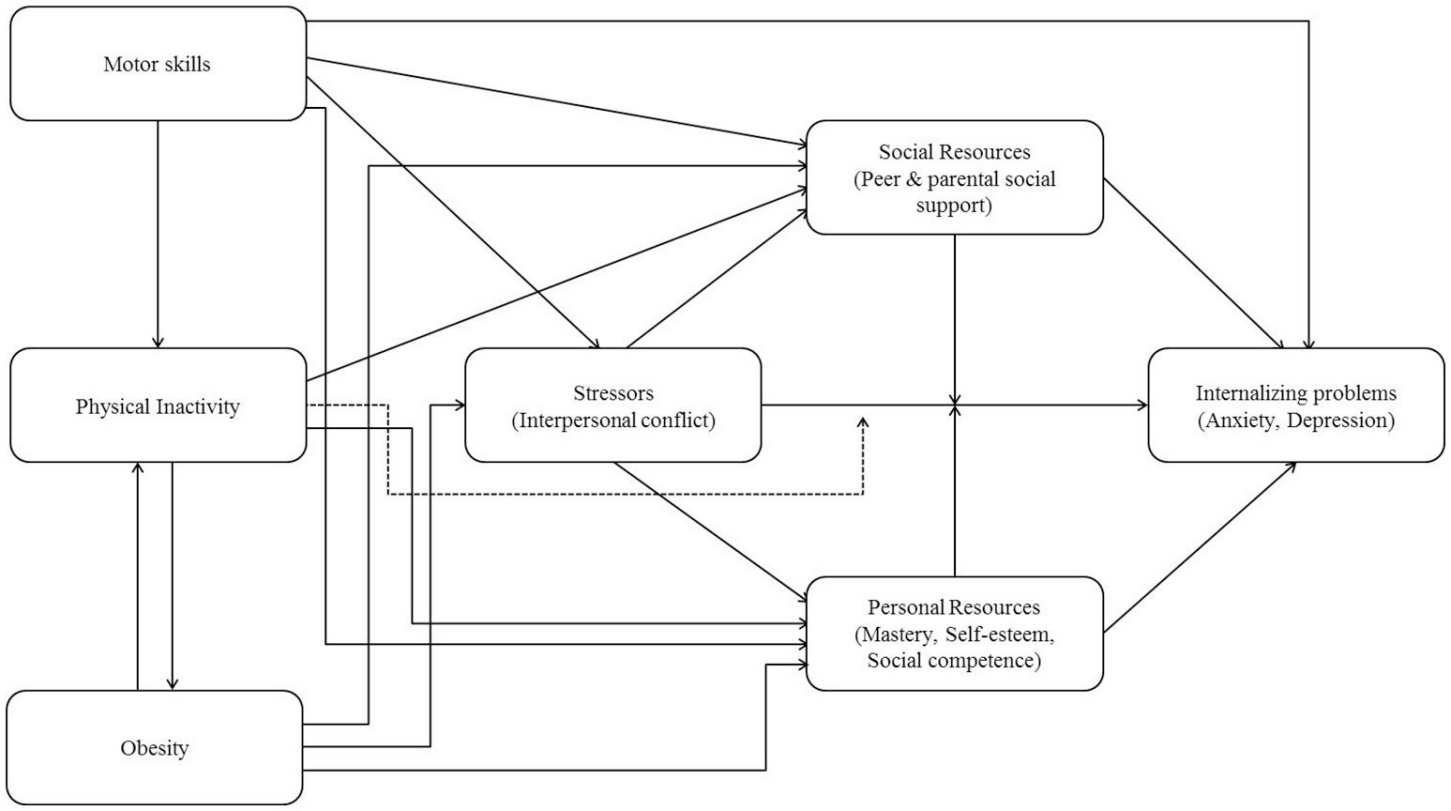
**The Environmental Stress Hypothesis framework as adapted by Mancini et al. (2016)**.

In terms of a more systematic understanding of the association between motor coordination problems and mental health, Mancini et al. (2016) recently adapted a conceptual framework termed the Environmental Stress Hypothesis (see Figure 1) in this journal. While the framework is based on Pearlin‘s stress process model (Pearlin et al., 1981; Pearlin, 1989), the term Environmental Stress Hypothesis was initially inducted by Cairney et al. (2010b) and elaborated by Cairney et al. (2013) in the context of Developmental Coordination Disorder (DCD; Blank et al., 2012).

Following the key-assumption of the framework, poor motor skills—in terms of observable motor coordination problems—are considered a primary source of stress which raises the risk for psychological distress via secondary environmental risk factors, so called stressors. Within the framework, psychological distress is represented in terms of internalizing problems. Longitudinal research documenting the impact of childhood motor coordination problems on internalizing problems in adulthood was recently provided by Poole et al. (2015); however, Cairney et al. (2013) state that the original framework could be applied for many different outcomes which presumably also accounts for the here discussed adaptation. Secondary environmental risk factors are defined as interpersonal conflicts with peers, teachers or parents. Corresponding cross-sectional research linking motor coordination problems with psychological distress via secondary environmental risk factors is provided by Wagner et al. (2012) who state that the relationship between DCD and internalizing as well as externalizing problems in school-aged children is at least partially mediated by peer problems. When focusing on the second part of the mediation, Mancini et al. (2016) assume the negative impact of interpersonal conflicts on psychological distress to be buffered by protective factors which they differentiate between social and personal resources. While personal resources include conceptions of mastery, self-esteem and social competence, social resources basically represent the social support provided by peers or parents. Concerning personal resources, recent intervention studies provide evidence that improving motor skills and participation has a positive impact on children's prosocial behavior (Piek et al., 2015) and that exercising in adolescents with low motor competencies fosters their physical self-perception (McIntyre et al., 2014). Concerning potential buffering effects, it is important to note that both resources are assumed to either mediate or moderate the risk factor-distress relation. To that effect, corresponding cross-sectional research indicates that the relationship between motor coordination and emotional well-being or emotional problems is fully mediated by self-perceptions of competence (Rigoli et al., 2012) and social skills (Wilson et al., 2013) or self-concept (Viholainen et al., 2014), respectively. Corresponding longitudinal research suggests that children with probable DCD are less likely to develop subsequent mental health problems in cases of higher verbal intelligence, self-esteem, academic performance, social communication skills and in the absence of bullying (Lingam et al., 2012). In addition to the core pathways of the stress process, Mancini et al. (2016) also include physical inactivity as well as obesity within their framework; both measures are ordered in a reciprocal deterministic relationship and are assumed to negatively affect the discussed intermediary pathways leading to psychological distress.

In accordance with Missiuna and Campbell (2014) we likewise greatly acknowledge the potential of the framework to develop our knowledge of the complex interrelation of factors putting children with motor coordination problems at greater risk for mental health problems. However, before being able to derive and explore respective interventional measures which could alter the assumed trajectories, one has to keep in mind that a comprehensive examination of all the relevant factors identified within the Environment Stress Hypothesis framework has yet to be conducted (Mancini et al., 2016). In this regard, Missiuna and Campbell (2014) postulate that “[…] prospective longitudinal research is needed, starting when children are young, before the psychological problems have emerged.” and that “[.] these studies require a population-based approach” (p. 127). Following the desideratum identified by Mancini et al. (2016) as well as the methodological postulate by Missiuna and Campbell (2014), the aim of this paper is to initially contribute to a population-based longitudinal elaboration of the Environmental Stress Hypothesis framework.

Referring to Mancini et al. (2016), existing (community-based) longitudinal research provides certain evidence for the causal assumptions within the framework during the course of childhood (Lingam et al., 2012) or from childhood to adulthood (Poole et al., 2015), respectively. However, most of the problems highlighted within the framework such as internalizing problems, physical inactivity or interpersonal conflicts apparently become crucial during the course of adolescence. We are therefore aiming to address a current research gap by investigating whether motor coordination problems in childhood actually have a direct impact on the development of psychological distress as well as on corresponding secondary risk and protective factors in adolescence.

Since motor coordination problems should not typically be diagnosed before 5 years of age (Blank et al., 2012, recommendation 8) we consider it useful to start the required population-based longitudinal research in elementary-school.

When defining the primary stressor within the Environmental Stress Hypothesis one must further consider, that following the International Classification of Diseases (ICD 10), motor coordination problems (F 0.82) could be categorized as either gross (F 82.0) or fine (F 82.1) motor dysfunctions (see also Blank et al., 2012, recommendation 5). While specific fine motor coordination problems may be more relevant for school achievement, gross motor coordination problems particularly seem to be important for participation and development of social contact with peers (see Blank et al., 2012; recommendation 19; statement 3). Since the Environmental Stress Hypothesis framework mainly addresses participation as well as the development of social contact with peers, the intended elaboration seems most promising under the category of gross motor coordination problems.

Finally, we propose to operationalize psychological distress as well as corresponding secondary risk and protective factors with reference to significant preliminary studies (e.g., Green et al., 2006; see Table 1) and opt for a binary encoding of both, the exposure (e.g., Skinner and Piek, 2001) as well as all respective outcomes with reference to common epidemiological practice (e.g., Turner et al., 2010).

**Table 1:** **Constructs, operationalization, and assignment to the hypotheses**.

**Constructs and exemplary operationalizations as used by Mancini et al. (2016)**	**Operationalizations in our study**	**Hypothesis**
Motor skills	Gross motor coordination problems	1
Physical Inactivity	Avoiding organized physical activities	2
Obesity	Elevated body mass	3
Stressors (Interpersonal conflict)	Peer problems	4
Personal Resources (Mastery, Self-esteem, Social competence)	Diminished prosocial behavior	5
Social Resources (Peer and parental social support)	Bonding with sedentary peers	6
Internalizing Problems (Anxiety, Depression)	Emotional problems	7
	Conduct problems^a^	8

a *Conduct problems were integrated as an important aspect of externalizing problems to foster an extended view on potential mental health outcomes*.

For answering the above stated research question and taking into account the age- and construct-related specifications as described above, it is assumed that elementary-school children with gross motor coordination problems show a higher risk of persistent gross motor coordination problems (Hypothesis 1), avoiding organized physical activities (Hypothesis 2), an elevated body mass (Hypothesis 3), peer problems (Hypothesis 4), diminished prosocial behavior (Hypothesis 5), bonding with sedentary peers (Hypothesis 6) as well as emotional (Hypothesis 7), and conduct (Hypothesis 8) problems in adolescence compared to elementary-school children without gross motor coordination problems.

## Materials and methods

### Participants

The here pursued elaboration of the Environmental Stress Hypothesis framework is based on a longitudinal sample of 940 participants who were (i) recruited as part of a population-based representative survey on health, physical fitness and physical activity in childhood and adolescence, (ii) assessed twice within 6 years, between the ages of 6 and 10 years old as well as between the ages of 12 and 16 years old (Response Rate: 55.9%) and (iii) classified as having gross motor coordination problems (*N* = 115) or having no gross motor coordination problems (*N* = 825) at baseline.

Baseline-data were obtained from the nationwide German Health Interview and Examination Survey for Children and Adolescents (KiGGS; www.kiggs.de) which was conducted by the Robert Koch-Institute (RKI, Berlin) between 2003 and 2006 (KiGGS Baseline Study; Kurth et al., 2008). The KiGGS Baseline Study included a core survey as well as five in-depth module studies carried out with corresponding KiGGS subsamples. One of those module studies—the Motorik-Modul (MoMo) Baseline Study—was conducted by the Karlsruhe Institute of Technology and provided nationwide representative data on the physical fitness and physical activity status of German children and adolescents. The RKI aimed to obtain a study sample that is representative of children and adolescents with primary residence in Germany for the KiGGS Baseline Study. Thus, the RKI and the Centre for Surveys, Methods and Analysis (GESIS) used a stratified multi-stage probability sample with three evaluation levels. First, a systematic sample of 167 primary sampling units was selected from an inventory of German communities (Kurth et al., 2008). Second, an age-stratified sample of randomly selected children and adolescents was drawn from the official registers of local residents with a total of 17,641 participants aged between 0 and 17 years old (Kamtsiuris et al., 2007) Third, 7866 children and adolescents aged between 4 and 17 years old from the KiGGS baseline sample were randomly assigned to the MoMo baseline sample of which 4529 children and adolescents in the same age range finally participated in the MoMo Baseline Study (response rate: 57.6%). To improve representativeness of the study results, deviations of the sample from the population structure regarding age, sex, region, and country of citizenship were corrected by weighing the data (Kamtsiuris et al., 2007). Depending on the pattern of the missingness, different methods (e.g., listwise deletion, multiple imputation, full information maximum likelihood) were applied. Results of the MoMo Baseline Study have been published in several consecutive research papers (e.g., Wagner et al., 2010; Tittlbach et al., 2011; Woll et al., 2011; Jekauc et al., 2012; Peterhans et al., 2013; Reimers et al., 2013; Spengler and Woll, 2013).

The MoMo Baseline Study continued longitudinally in 2009 as a joint project between the University of Konstanz, the Karlsruhe Institute of Technology and the University of Education Karlsruhe (see Wagner et al., 2014) parallel to the longitudinal continuation of the KiGGS Baseline Study (Hölling et al., 2012). The sub-sample structure was maintained so that each member of the MoMo cohort also belongs to the KiGGS cohort. The first follow-up of the MoMo Longitudinal study began in September 2009 and ended in July 2012 with two subsequent survey waves to be conducted between 2014 and 2016 and between 2018 and 2020, respectively. The MoMo Longitudinal sample (Baseline to first follow-up) included 2178 participants aged between 10 and 23 years old at first which equals an overall response rate of 48.1%. For 664 participants of the longitudinal sample who were unable to attend any of the test dates, at least physical activity was assessed via questionnaire which raised the corresponding response rate up to 62.8% (total longitudinal sample size: 2842 participants).

Within this paper we focus on elementary-school children between the ages of 6 and 10 years old at baseline (*N* = 1681; *M*_age_ = 8.27 ± 1.48; 50.4% boys) who were re-examined in adolescence between the ages of 12 and 16 years old (*N* = 940; Response Rate: 55.9%; *M*_age_ = 14.37 ± 1.46 years; 49.1% boys). Participants in the longitudinal sample were classified according to their gross motor coordination status (gross motor coordination problems/no gross motor coordination problems) at baseline (elementary-school age) using three common gross motor coordination tasks. A description of corresponding tasks as well as their composition to the respective gross motor coordination score is provided at the beginning of the *measures* section. Table 2 shows the sociodemographic characteristics of the longitudinal sample including participants mean age as well as the distribution of gender, migration background (Kurth et al., 2008) and socioeconomic status (Winkler and Stolzenberg, 1999) differed by study group and survey wave.

**Table 2:** **Sociodemographic characteristics of the longitudinal sample (*N* = 940)**.

	**Total**		**Age in elementary-school**		**Age in adolescence**		**Boys**		**Girls**		**No migration background**		**Migration background**		**High SES**		**Middle SES**		**Low SES**	
	***N***	***%***	***M***	***SD***	***M***	***SD***	***N***	***%***	***N***	***%***	***N***	***%***	***N***	***%***	***N***	***%***	***N***	***%***	***N***	***%***
GMCP in elementary-school	115	12.2	8.12	1.53	14.35	1.52	55	47.8	60	52.2	105	92.1	9	7.9	27	23.5	58	50.4	30	26.1
No GMCP in elementary-school	825	87.8	8.15	1.45	14.38	1.45	407	49.3	418	50.7	776	94.7	43	5.3	266	32.2	428	51.9	131	15.9
Total	940	100.0	8.14	1.46	14.37	1.46	462	49.15	478	50.85	881	94.4	52	5.6	293	31.2	486	51.7	161	17.1

*GMCP, Gross motor coordination problems; SES, Socioeconomic Status*.

Study groups did not significantly differ by age, neither at baseline [*F*_(1, 938)_ = 0.03, *p* = 0.867, $\eta_{p}^{2}$ = 0.000] nor at the time of the first follow up [*F*_(1, 938)_ = 0.04, *p* = 0.851, $\eta_{p}^{2}$ = 0.000]. Further, no significant difference was found concerning the distribution of gender [χ^2^_(1, *N* = 940)_ = 0.09, *p* = 0.762, Φ = 0.010] or migration background [χ^2^_(1, *N* = 933)_ = 1.33, *p* = 0.249, Φ = 0.038] among both study groups, respectively. However, there was a significant but small difference concerning the distribution of socioeconomic status (SES) at baseline among both study groups [χ^2^_(2, *N* = 940)_ = 8.67, *p* < 0.05; *Cramér's V* = 0.096] with a comparatively higher proportion of low SES within the group of children with gross motor coordination problems. Compared to the representative baseline sample, our longitudinal sample provides slightly more high SES elementary-school children (31.2 vs. 26.3%) indicating an expectable selection bias.

To further verify the distinction between both study groups with reference to Dewey et al. (2002), we not only compared elementary-school children with and without gross motor coordination problems regarding their gross motor coordination score, but also regarding their Attention Deficit Hyperactivity Disorder (ADHD; psychological assessment) and language development (LD; speech therapist assessment) status (see Table 3).

**Table 3:** **Characteristics of gross motor coordination performance and DCD-related co-morbidities within the longitudinal sample (*N* = 940)**.

	**Gross motor coordination in elementary-school [Z-Score]**			**Gross motor coordination in adolescence [Z-Score]**			**No ADHD in elementary-school**		**ADHD in elementary-school**		**No delayed language development in elementary-school**		**Delayed language development in elementary-school**	
	***N***	***M***	***SD***	***N***	***M***	***SD***	***N***	***%***	***N***	***%***	***N***	***%***	***N***	***%***
GMCP in elementary-school	115	−3.67	1.23	115	−2.50	2.57	101	92.7	8	7.3	61	91.0	6	9.0
No GMCP in elementary-school	825	0.71	1.75	825	0.35	2.05	774	97.0	24	3.0	453	96.6	16	3.4
Total	940	0.17	2.22	940	0	2.31	875	96.5	32	3.5	514	95.9	22	4.1

*GMCP, Gross motor coordination problems; ADHD, Attention Deficit Hyperactivity Disorder*.

When compared to elementary-school children without gross motor coordination problems, elementary-school children with gross motor coordination problems not only showed a significantly lower gross motor coordination score at baseline [*F*_(1, 938)_ = 670.18, *p* < 0.001, $\eta_{p}^{2}$ = 0.417] but also 6 years later, at the time of the first follow up [*F*_(1, 938)_ = 183.51, *p* < 0.001, $\eta_{p}^{2}$ = 0.164]. Further, there was a significant but small difference concerning the distribution of ADHD [χ^2^_(1, *N* = 907)_ = 5.29, *p* < 0.05, Φ = 0.076] and LD [χ^2^_(1, *N* = 536)_ = 4.58, *p* < 0.05, Φ = 0.092] status among both study groups with a slightly higher proportion of ADHD and delayed LD within the group of elementary-school children with gross motor coordination problems.

### Measures

A comprehensive list of all concepts and measures used within the MoMo Longitudinal study can be found in Wagner et al. (2014). Subsequent description of measures (in overview see Table 4) is limited to those relevant for the here pursued elaboration of the Environmental Stress Hypothesis framework; categorization of raw data was processed using SPSS 23.0 (Arbuckle, 2014).

**Table 4:** **Operationalizations, measures and references**.

**Operationalizations**	**Measures**	**References**
Gross motor coordination problems	MoMo test battery	Worth et al., 2015
Avoiding organized physical activities	MoMo-Physical Activity Questionnaire	Jekauc et al., 2013c
Bonding with sedentary peers		
Elevated body mass	Body-Mass Index	Stolzenberg et al., 2007
Peer problems	Strength and Difficulties Questionnaire	Goodman, 1997
Diminished prosocial behavior		
Emotional problems		
Conduct problems		

**Figure 2:**
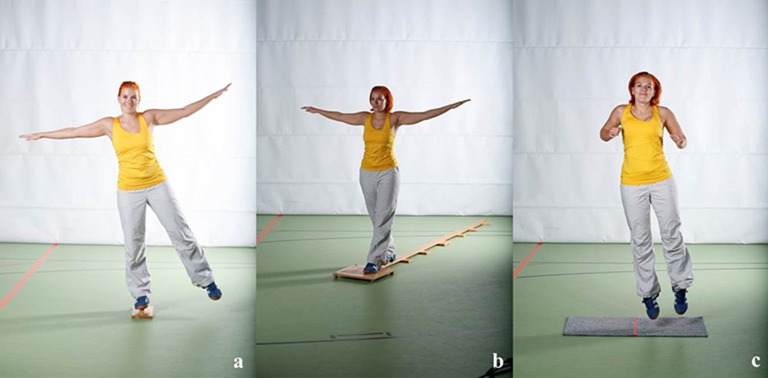
**Gross motor coordination tasks** (**a**, one-leg stand; **b**, balancing backwards; **c**, jumping side-to-side).

Gross motor coordination problems were assessed using three common gross motor coordination tasks from the MoMo test battery (Wort het al., 2015). In particular, participants were asked to stand on their dominant leg for 60 s (Schilling and Baedke, 1980), to balance backwards on three bars of different widths (Schilling, 1974) and to perform as many side-to-side jumps as possible within 15 s on a small carpet-mat (Schilling, 1974; see Figure 2).

All motor tests as described above have already been successfully applied to our baseline data (e.g., Woll et al., 2013) and correspond with selected items included in DCD-specific test batteries such as the Movement Assessment Battery for Children (M-ABC; Smits-Engelsman et al., 1998; Henderson et al., 2007) or the Bruininks-Oseretsky Test of Motor Proficiency (BOT, Bruininks and Bruininks, 2014; Fransen et al., 2014). For each gross motor coordination task we calculated age- and gender-specific *z*-scores (independently for both survey waves in terms of Hypothesis 1) and combined the standardized scores to a gross motor coordination score. Respective gross motor coordination score covers two dimensions: gross motor coordination under time pressure (jumping side-to-side) as well as under precision pressure (one-leg stand; balancing backwards; e.g., Lämmle et al., 2010) which is represented by an expectable diminished internal consistency (*Cronbach‘s* α = 0.62). Following common practice in defining a group with significant motor problems (see Cairney et al., 2013) we calculated the age- and gender-specific 15th Percentile of the respective gross motor coordination score and used this cut-off value to classify each participant as either having (≤15th Percentile) or not having (>15th Percentile) gross motor coordination problems.

Avoiding organized physical activities (Hypothesis 2) as well as bonding with sedentary peers (Hypothesis 6) were assessed via self-report using the MoMo-Physical Activity Questionnaire (MoMo-PAQ) which was found to be a reliable and valid assessment with psychometric properties comparable to other established physical activity questionnaires (Jekauc et al., 2013c). In particular, we asked the participants “Are you currently member of a sports club? (Yes/No)” or “How many of your friends are physically active on a regular basis? (Not any or a few/some or most),” respectively. Hereby, currently not being member of a sports club or not having any or only a few active friends was considered an indication for avoiding organized physical activities or bonding with sedentary peers, respectively.

An elevated body mass (Hypothesis 3) was determined on the basis of an independent measurement of participants' weight and height and the subsequent calculation of their individual Body-Mass-Index (BMI; Stolzenberg et al., 2007) which was found to be the best respective monitoring tool (Hall and Cole, 2006). Using the German BMI-cut-off values by Kromeyer-Hauschild et al. (2011), we classified participants as either having (>90th Percentile; overweight and obesity) or not having (<90th Percentile; normal weight) an elevated body mass.

Both, the MoMo-PAQ as well as the BMI have already been successfully applied to our longitudinal data (Spengler et al., 2014; Rauner et al., 2015).

Finally, peer problems (Hypothesis 4), diminished prosocial behavior (Hypothesis 5) as well emotional (Hypothesis 7) and conduct (Hypothesis 8) problems were assessed using the parent version of the Strength and Difficulties Questionnaire (SDQ, Goodman, 1997) in both survey waves which was found to be a valid and helpful instrument in the epidemiological context (Rothenberger et al., 2008). On the basis of the German SDQ-cut-off points (www.sdq.org; Woerner et al., 2002) and following the binary encoding used by Goodman et al. (2000), we classified participants as either having (>90th Percentile; abnormal) or not having (≤90 Percentile; borderline and normal) peer, emotional or conduct problems or diminished prosocial behavior, respectively.

All motor tests (including the measurement of weight and height) as well as the MoMo-PAQ were guided by experienced assessors of the MoMo-team in the respective test-centers in both survey waves and took each participant between 70 and 90 min to complete it. The SDQ was guided by experienced assessors of the KiGGS-team in the respective test-centers at baseline and within a telephone interview at the time of the first follow-up.

Participants' testing and questioning was approved in written form by the ethical commission of the involved universities and research centers.

### Statistical analysis

For analyzing the developmental risk of having gross motor coordination problems concerning the binary encoded outcome measures as described within the respective section, we opted for binary logistic regressions (e.g., Bender, 2009) using SPSS 23.0 (Arbuckle, 2014). Taking into account the methodological shortcomings of recent longitudinal studies with reference to the Environmental Stress Hypothesis framework (see Mancini et al., 2016), we included the baseline value of the respective dependent variable as a primary predicting variable in order to control for its stability. Furthermore, following the results provided by Woll et al. (2013), we integrated participants' age as a continuous variable at baseline as well as participants sex as co-variates within our binary logistic models. The significance level for all statistical tests was set a priori to α = 0.05 to control for type I error; one-tailed testing was performed to determine the impact of gross motor coordination problems given the fact that all respective alternative hypotheses were directed (see Gravetter and Wallnau, 2014).

## Results

### Persistence of gross motor coordination problems (Hypothesis 1)

It was assumed that elementary-school children with gross motor coordination problems show a higher risk of persistent gross motor coordination problems in adolescence compared to elementary-school children without gross motor coordination problems (Hypothesis 1); testing of corresponding null-hypothesis was based on a total of 940 longitudinal observations.

Descriptive results indicated that 47.8% (*n* = 55) of elementary-school children with gross motor coordination problems compared to 10.3% (*n* = 85) of elementary-school children without gross motor coordination problems show gross motor coordination problems in adolescence; in line with hypothesis 1, the hereon based results of the binary logistic regression (see Table 5) indicated that elementary-school children with gross motor coordination problems show a 7.99 times higher risk (*p* < 0.001) of gross motor coordination problems in adolescence compared to elementary-school children without gross motor coordination problems. Furthermore, analysis of integrated co-variates indicated that elementary-school children's age (*OR* = 1.01, *p* = 0.892) and elementary-school children's sex (*OR* = 0.94, *p* = 0.731) both show no significant impact on the risk of gross motor coordination problems in adolescence.

**Table 5:** **Binary logistic regression to determine the impact of gross motor coordination problems in childhood on gross motor coordination problems in adolescence**.

	**Gross motor coordination problems in adolescence**				
	***B***	***Wald***	***df***	***p***	***OR***
GMCP^a^	2.08	89.89	1	0.000^c^	7.99
Age at baseline	0.01	0.02	1	0.892	1.01
Sex^b^	−0.07	0.12	1	0.731	0.94
Intercept	−2.13	12.56	1	0.000	0.12
Nagelkerke‘s Pseudo-*R*^2^					0.152
*N*					940

*GMCP, Gross motor coordination problems*;

a *Reference, no GMCP in childhood*;

b *Reference, boys*;

c *One-tailed*.

### Gross motor coordination problems and avoiding organized physical activities (Hypothesis 2)

It was assumed that elementary-school children with gross motor coordination problems show a higher risk of *avoiding* organized physical activities in adolescence compared to elementary-school children without gross motor coordination problems (Hypothesis 2); testing of corresponding null-hypothesis was based on a total of 913 longitudinal observations.

Descriptive results indicated that 45% (*n* = 50) of elementary-school children with gross motor coordination problems compared to 31.3% (*n* = 251) of elementary-school children without gross motor coordination problems avoid organized physical activities in adolescence; in line with hypothesis 2, the hereon based results of the binary logistic regression (see Table 6) indicated that elementary-school children with gross motor coordination problems show a 1.53 times higher risk (*p* < 0.05) of avoiding organized physical activities in adolescence compared to elementary-school children without gross motor coordination problems. Furthermore, analysis of integrated co-variates indicated that elementary-school children who avoid organized physical activities show a 4.44 times higher risk (*p* < 0.001), older elementary-school children show a 1.13 times higher risk (*p* < 0.05) and girls show a 1.36 times higher risk (*p* < 0.05) of *avoiding* organized physical activities in adolescence compared to elementary-school children who do not avoid organized physical activities, younger elementary-school children and boys, respectively.

**Table 6:** **Binary logistic regression to determine the impact of gross motor coordination problems in childhood on avoiding organized physical activities in adolescence**.

	**Avoiding organized physical activities in adolescence**				
	***B***	***Wald***	***df***	***p***	***OR***
GMCP^a^	0.43	3.71	1	0.027^d^	1.53
Avoiding organized physical activities in childhood^b^	1.49	92.81	1	0.000	4.44
Age at baseline	0.12	5.43	1	0.020	1.13
Sex^c^	0.30	3.95	1	0.047	1.36
Intercept	−2.75	32.75	1	0.000	0.06
Nagelkerke‘s Pseudo-*R*^2^					0.172
*N*					913

*GMCP, Gross motor coordination problems*;

a *Reference, no GMCP in childhood*;

b *Reference, not avoiding organized physical activities in childhood*;

c *Reference, boys*;

d *One-tailed*.

### Gross motor coordination problems and an elevated body mass (Hypothesis 3)

It was assumed that elementary-school children with gross motor coordination problems show a higher risk of an elevated body mass in adolescence compared to elementary-school children without gross motor coordination problems (Hypothesis 3); testing of corresponding null-hypothesis was based on a total of 939 longitudinal observations.

Descriptive results indicated that 27.8% (*n* = 32) of elementary-school children with gross motor coordination problems compared to 12.3% (*n* = 101) of elementary-school children without gross motor coordination problems show an elevated body mass in adolescence; in line with hypothesis 3, the hereon based results of the binary logistic regression (see Table 7) indicated that elementary-school children with gross motor coordination problems show a 1.78 times higher risk (*p* < 0.05) of an elevated body mass in adolescence compared to elementary-school children without gross motor coordination problems. Furthermore, analysis of integrated co-variates indicated that elementary-school children with an elevated body mass show a 17.22 times higher risk (*p* < 0.001) and girls show a 1.85 times lower risk (1/0.54; *p* < 0.01) of an elevated body mass in adolescence compared to normal-weighed elementary-school children and boys, respectively, whereat elementary-school children‘s age had no significant impact (*OR* = 1.04, *p* = 0.618) on the risk of developing an elevated body mass in adolescence.

**Table 7:** **Binary logistic regression to determine the impact of gross motor coordination problems in childhood on an elevated body mass in adolescence**.

	**Elevated body mass in adolescence**				
	***B***	***Wald***	***df***	***p***	***OR***
GMCP^a^	0.58	4.25	1	0.020^d^	1.78
Elevated body mass in childhood^b^	2.85	116.29	1	0.000	17.22
Age at baseline	0.04	0.25	1	0.618	1.04
Sex^c^	−0.61	7.95	1	0.005	0.54
Intercept	−1.76	7.46	1	0.006	0.17
Nagelkerke‘s Pseudo-*R*^2^					0.255
*N*					939

*GMCP, Gross motor coordination problems*;

a *Reference, no GMCP in childhood*;

b *Reference, no elevated body mass in childhood*;

c *Reference, boys*;

d *One-tailed*.

### Gross motor coordination problems and peer problems (Hypothesis 4)

It was assumed that elementary-school children with gross motor coordination problems show a higher risk of peer problems in adolescence compared to elementary-school children without gross motor coordination problems (Hypothesis 4); testing of corresponding null-hypothesis was based on a total of 937 longitudinal observations.

Descriptive results indicated that 15.8% (*n* = 18) of elementary-school children with gross motor coordination problems compared to 9.5% (*n* = 78) of elementary-school children without gross motor coordination problems show peer problems in adolescence; contrary to hypothesis 4, the hereon based results of the binary logistic regression (see Table 8) indicated that elementary-school children with gross motor coordination problems do not show a significantly higher risk (*OR* = 1.35, *p* = 0.164) of peer problems in adolescence compared to elementary-school children without gross motor coordination problems. Furthermore, analysis of integrated co-variates indicated that elementary-school children with peer problems show a 4.80 times higher risk (*p* < 0.001) and girls show a 1.67 times lower risk (1/0.60; *p* < 0.05) risk of peer problems in adolescence compared to elementary-school children without peer problems and boys, whereat elementary-school children's age had no significant impact (*OR* = 0.95, *p* = 0.515) on the risk of peer problems in adolescence.

**Table 8:** **Binary logistic regression to determine the impact of gross motor coordination problems in childhood on peer problems in adolescence**.

	**Peer problems in adolescence**				
	***B***	***Wald***	***df***	***p***	***OR***
GMCP^a^	0.30	0.96	1	0.164^d^	1.35
Peer problems in childhood^b^	1.57	29.87	1	0.000	4.80
Age at baseline	−0.05	0.42	1	0.515	0.95
Sex^c^	−0.51	5.14	1	0.023	0.60
Intercept	−1.30	3.67	1	0.056	0.27
Nagelkerke‘s Pseudo-*R*^2^					0.079
*N*					937

*GMCP, Gross motor coordination problems*;

a *Reference, no GMCP in childhood*;

b *Reference, no peer problems in childhood*;

c *Reference, boys*;

d *One-tailed*.

### Gross motor coordination problems and diminished prosocial behavior (Hypothesis 5)

It was assumed that elementary-school children with gross motor coordination problems show a higher risk of diminished prosocial behavior in adolescence compared to elementary-school children without gross motor coordination problems (Hypothesis 5); testing of corresponding null-hypothesis was based on a total of 937 longitudinal observations.

Descriptive results indicated that 2.6% (*n* = 3) of elementary-school children with gross motor coordination problems compared to 1.3% (*n* = 11) of elementary-school children without gross motor coordination problems show diminished prosocial behavior in adolescence; contrary to hypothesis 5, the hereon based results of the binary logistic regression (see Table 9) indicated that elementary-school children with gross motor coordination problems do not show a significantly higher risk (*OR* = 1.90, *p* = 0.168) of diminished prosocial behavior in adolescence compared to elementary-school children without gross motor coordination problems. Furthermore, analysis of integrated co-variates indicated that elementary-school children with diminished prosocial behavior show a 7.38 times higher risk (*p* < 0.05) of diminished prosocial behavior in adolescence compared to elementary-school children with a normal prosocial behavior, whereat both elementary-school children's age (*OR* = 0.93, *p* = 0.694) and sex (*OR* = 0.43, *p* = 0.163) had no significant impact on the risk of diminished prosocial behavior in adolescence.

**Table 9:** **Binary logistic regression to determine the impact of gross motor coordination problems in childhood on diminished prosocial behavior in adolescence**.

	**Diminished prosocial behavior in adolescence**				
	***B***	***Wald***	***df***	***p***	***OR***
GMCP^a^	0.64	0.09	1	0.168^d^	1.90
Diminished prosocial behavior in childhood^b^	2.00	5.90	1	0.015	7.38
Age at baseline	−0.08	0.16	1	0.694	0.93
Sex^c^	−0.84	1.95	1	0.163	0.43
Intercept	−2.67	2.55	1	0.111	0.07
Nagelkerke‘s Pseudo-*R*^2^					0.060
*N*					937

*GMCP, Gross motor coordination problems*;

a *Reference, no GMCP in childhood*;

b *Reference, no diminished prosocial behavior in childhood*;

c *Reference, boys*;

d *One-tailed*.

### Gross motor coordination problems and bonding with sedentary peers (Hypothesis 6)

It was assumed that elementary-school children with gross motor coordination problems show a higher risk of bonding with sedentary peers in adolescence compared to elementary-school children without gross motor coordination problems (Hypothesis 6); testing of corresponding null-hypothesis was based on a total of 817 longitudinal observations.

Descriptive results indicated that 36.3% (*n* = 37) of elementary-school children with gross motor coordination problems compared to 21.1% (*n* = 151) of elementary-school children without gross motor coordination problems are bonding with sedentary peers in adolescence; in line with hypothesis 6, the hereon based results of the binary logistic regression (see Table 10) indicated that elementary-school children with gross motor coordination problems show a 1.84 times higher risk (*p* < 0.01) of bonding with sedentary peers in adolescence compared to elementary-school children without gross motor coordination problems. Furthermore, analysis of integrated co-variates indicated that elementary-school children who are bonding with sedentary peers show a 1.92 times higher risk (*p* < 0.01) and girls show a 2.64 times higher risk (*p* < 0.001) of bonding with sedentary peers in adolescence compared to elementary-school children who are bonding with physically active peers and boys, whereby elementary-school children‘s age had no significant impact (*OR* = 1.04, *p* = 0.475) on the risk of bonding with sedentary peers in adolescence.

**Table 10:** **Binary logistic regression to determine the impact of gross motor coordination problems in childhood on bonding with sedentary peers in adolescence**.

	**Bonding with sedentary peers in adolescence**				
	***B***	***Wald***	***df***	***p***	***OR***
GMCP^a^	0.61	6.64	1	0.005^d^	1.84
Bonding with sedentary peers in childhood^b^	0.65	9.80	1	0.002	1.92
Age at baseline	0.04	0.51	1	0.475	1.04
Sex^c^	0.97	29.51	1	0.000	2.64
Intercept	−3.28	35.05	1	0.000	0.04
Nagelkerke‘s Pseudo-*R*^2^					0.095
*N*					817

*GMCP, Gross motor coordination problems*;

a *Reference, no GMCP in childhood*;

b *Reference, bonding with physically active peers in childhood*;

c *Reference, boys*;

d *One-tailed*.

### Gross motor coordination problems and emotional problems (Hypothesis 7)

It was is assumed that elementary-school children with gross motor coordination problems show a higher risk of emotional problems in adolescence compared to elementary-school children without gross motor coordination problems (Hypothesis 7); testing of corresponding null-hypothesis was based on a total of 937 longitudinal observations.

Descriptive results indicated that 16.7% (*n* = 19) of elementary-school children with gross motor coordination problems compared to 9.5% (*n* = 78) of elementary-school children without gross motor coordination problems show emotional problems in adolescence; in line with hypothesis 7, the hereon based results of the binary logistic regression (see Table 11) indicated that elementary-school children with gross motor coordination problems show a 1.73 times higher risk (*p* < 0.05) of emotional problems in adolescence compared to elementary-school children without gross motor coordination problems. Furthermore, analysis of integrated co-variates indicated that elementary-school children with emotional problems show a 4.61 times higher risk (*p* < 0.001) of emotional problems in adolescence compared to elementary-school children without emotional problems, whereat both children‘s age (*OR* = 0.97, *p* = 0.661) and sex (*OR* = 1.36, *p* = 0.169) had no significant impact on the risk of emotional problems in adolescence.

**Table 11:** **Binary logistic regression to determine the impact of gross motor coordination problems in childhood on emotional problems in adolescence**.

	**Emotional problems in adolescence**				
	***B***	***Wald***	***df***	***p***	***OR***
GMCP^a^	0.55	3.61	1	0.029^d^	1.73
Emotional problems in childhood^b^	1.53	29.22	1	0.000	4.61
Age at baseline	−0.03	0.19	1	0.661	0.97
Sex^c^	0.31	1.89	1	0.169	1.36
Intercept	−2.66	14.78	1	0.000	0.07
Nagelkerke‘s Pseudo-*R*^2^					0.071
*N*					937

*GMCP, Gross motor coordination problems*;

a *Reference, no GMCP in childhood*;

b *Reference, no emotional problems in childhood*;

c *Reference, boys*;

d *One-tailed*.

### Gross motor coordination problems and conduct problems (Hypothesis 8)

It was assumed that elementary-school children with gross motor coordination problems show a higher risk of conduct problems in adolescence compared to elementary-school children without gross motor coordination problems (Hypothesis 8); testing of corresponding null-hypothesis was based on a total of 937 longitudinal observations.

Descriptive results indicated that 19.3% (*n* = 22) of elementary-school children with gross motor coordination problems compared to 12.2% (*n* = 100) of elementary-school children without gross motor coordination problems show conduct problems in adolescence; in line with hypothesis 8, the hereon based results of the binary logistic regression (see Table 12) indicated that elementary-school children with gross motor coordination problems show a 1.79 times higher risk (*p* < 0.05) of conduct problems in adolescence compared to elementary-school children without gross motor coordination problems. Furthermore, analysis of integrated co-variates indicated that elementary-school children with conduct problems show a 8.38 times higher risk (*p* < 0.001) and older elementary-school children show a 1.28 times lower risk (1/0.78; *p* < 0.01) of conduct problems in adolescence compared to elementary-school children without conduct problems and younger elementary-school children, whereat children‘s sex had no significant impact (*OR* = 0.77, *p* = 0.217) on the risk of conduct problems in adolescence.

**Table 12:** **Binary logistic regression to determine the impact of gross motor coordination problems in childhood on conduct problems in adolescence**.

	**Conduct problems in adolescence**				
	***B***	***Wald***	***df***	***p***	***OR***
GMCP^a^	0.58	4.24	1	0.020^d^	1.79
Conduct problems in childhood^b^	2.13	89.16	1	0.000	8.38
Age at baseline	−0.24	10.28	1	0.001	0.78
Sex^c^	−0.26	1.52	1	0.217	0.77
Intercept	−0.28	0.19	1	0.67	0.76
Nagelkerke‘s Pseudo-*R*^2^					0.195
*N*					937

*GMCP, Gross motor coordination problems*;

a *Reference, no GMCP in childhood*;

b *Reference, no conduct problems in childhood*;

c *Reference, boys*;

d *One-tailed*.

## Discussion

### Summary

**Figure 3:**
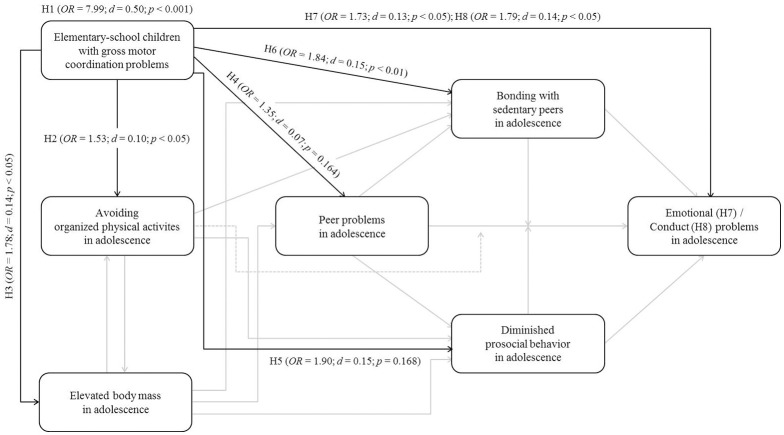
**Elaborated pathways within the Environmental Stress Hypothesis framework (*N* = 940; Baseline: 6–10 years; Follow-up: 12–16 years); H, Hypothesis; H1 refers to the persistence of gross motor coordination problems from elementary-school to adolescence**.

The Environmental Stress Hypothesis represents a heuristic framework recently adapted by Mancini et al. (2016). Within the framework poor motor skills—in terms of observable motor coordination problems—are considered a primary source of stress which raises the risk for psychological distress (e.g., internalizing problems) via secondary environmental risk factors (e.g., interpersonal conflicts with peers). Corresponding mediations are assumed to be moderated by social (e.g., parental support) and personal (e.g., social competence) resources as well as obesity-related physical inactivity. The aim of this paper was to contribute to a population-based longitudinal elaboration of the Environmental Stress Hypothesis framework by testing eight particular hypotheses addressing the direct impact of gross motor coordination problems in elementary-school on selected physical, behavioral and psychosocial outcomes in adolescence. Corresponding results are summarized in Figure 3.

In accordance with Mancini et al. (2016), results (see Figure 3) indicated that elementary-school children with gross motor coordination problems show a significantly higher risk of persistent gross motor coordination problems (Hypothesis 1), avoiding organized physical activities (Hypothesis 2), an elevated body mass (Hypothesis 3), bonding with sedentary peers (Hypothesis 6) as well as emotional (Hypothesis 7) and conduct problems (Hypothesis 8) in adolescence compared to elementary-school children without gross motor coordination problems. In contrast to Mancini et al. (2016), elementary-school children with gross motor coordination problems did not show a significantly higher risk of peer problems (Hypothesis 4) or diminished prosocial behavior (Hypothesis 5) in adolescence, respectively compared to elementary-school children without gross motor coordination problems; however, both effects were in the assumed direction. In extension to corresponding and recently published empirical research (in overview Mancini et al., 2016), this study is the first to provide population-based longitudinal data ranging from childhood to adolescence in the context of the Environmental Stress Hypothesis which can be considered a substantial methodological progress. Moreover, we integrated the stability of each respective dependent variable within our analysis allowing for a more detailed evaluation of the particular explanatory power of gross motor coordination problems over time. To that extent and by transferring the relative risks to more common effect sizes such as Cohen‘s *d* (see Figure 3), it is becoming evident that gross motor coordination problems represent a rather stable phenomenon and that their predictive power concerning the addressed physical, behavioral and psychosocial outcomes in adolescence is comparatively low. However, when interpreting our results, one has to be aware of several limitations described in the following section.

### Limitations

The 6-year time-interval between the baseline assessment and the first follow-up certainly provides a rather rough reflection of the developmental changes characterizing the transition from childhood to adolescence. In other words, the apparently low predictive power of gross motor coordination problems cannot be seen independently from our particular design and should therefore not be misunderstood in terms of a generalized weakening of the Environmental Stress Hypothesis framework.

The framework itself was originally developed for children with DCD. However, in our study we focused particularly on children's gross motor coordination performance rather than on the full spectrum of diagnostic DCD-criteria, did not apply recommended test batteries such as the M-ABC or the BOT and used the 15th percentile as a rather moderate cut-off for the distinction between children with and without gross motor coordination problems. Therefore, our study group with gross motor coordination problems apparently represents a superset of children including those with actual DCD. Thus, from a population-standpoint and despite the fact that we used a dichotomous sample-classification, our results provide certain evidence for the Environmental Stress hypothesis framework in terms of the recent Mancini et al. (2016) adaptation but may be different when explicitly focusing on children with DCD as originally intended by Cairney et al. (2013). Nonetheless, from an assessment standpoint it is fair criticism to state that our results are based only on three rather foundational motor skill tasks and thus, do not allow for conclusions on the evidence of the Environmental Stress Hypothesis framework across a broader spectrum of different motor skills.

Focusing on organized physical activities certainly excludes the possibility, that children and adolescents in our study could have also been active in other informal or school-related settings at the time of their questioning. Thus, our results on the linkage between gross motor coordination problems and physical inactivity are actually limited to the sports club setting or even more specifically, to participants' respective member status. In other words, results might display in a different manner when focusing on the amount of physical activity in this particular setting or when applying an extended setting-approach. Furthermore, while sports clubs certainly represent an important setting for sports and physical activity in Germany with a membership-rate of 57.4% in childhood and adolescence and an average exercise-rate of 4 h per week with moderate to high intensity (see Jekauc et al., 2013a), this may not be the case in other countries; therefore, our elaboration of the Environmental Stress Hypothesis framework apparently suffers from a lack of cross-cultural validity at this point.

Using the 90th BMI Percentile only provides evidence for the assumption that gross motor coordination problems have an impact on an elevated body-mass in general. Thus, one has to be aware of the fact that results may be different when explicitly focusing on the risk for pathological obesity as addressed by Mancini et al. (2016). Furthermore, we have to state that BMI provides a rather rough estimation of participants' total body fat and that respective German cut-off values slightly differ from the international standard provided by Cole et al. (2000) which limits the validity of our findings in several ways.

Concerning the assessment of psychological distress as well as corresponding secondary risk and protective factors we have to keep in mind that the *Strength and Difficulties Questionnaire* only provides screening information which cannot be equated with a respective clinical diagnosis. Thus, we might have indicated children and adolescents as having respective problems even though they would potentially not meet more restrictive clinical criteria. All the more when considering that our classification beyond the borderline cases followed the original SDQ cut-off recommendations whereat more recently, a dichotomous categorization has been proposed to further differentiate the so called *abnormal* category. Moreover, it has to be considered that parents of pubescent and especially conspicuous adolescents might not have sufficient emotional access to their children and thus, our informants were potentially not able to provide a valid personality profile at the time of the first follow-up. Concerning potential mental health problems it has to be stated that we only screened for emotional and conduct problems which certainly represent important aspects of internalizing and externalizing problems in terms of Mancini et al. (2016) but cannot be fully equated. This also accounts for the prosocial behavior scale as one important representative of personal resources or the assessment of peer sedentariness in the context of social resources. Furthermore, we do not want to leave unsaid that the low predictive power of gross motor coordination problems concerning the SDQ-subscales may also partially be due to possible mode effects (written questionnaire at baseline vs. telephone interview at the time of the first follow-up; Hölling et al., 2014).

Finally, it has to be stated that we initially tested direct pathways leading from gross motor coordination problems to the respective physical, behavioral and psychosocial outcomes. Thus, our results do not allow for an evaluation of the Environmental Stress Hypothesis framework in terms of the postulated mediating and moderating effects.

The limitations discussed in this section will be considered core elements of the following implications.

### Implications

Stronger evidence for the particular pathways within the Environmental Stress Hypothesis framework requires closed meshed monitoring (e.g., Cairney et al., 2010b). Designs such as the one used within the currently conducted *Coordination and Activity Tracking in CHildren* (CATCH) study (Cairney et al., 2015) are promising and should be considered for future research.

When aiming to further elaborate the Environmental Stress Hypothesis framework in terms of Mancini et al. (2016), one has to keep in mind that the gross motor coordination skills used in our study allow for the inference on rather foundational areas of movement (e.g., balance/postural control) which presumably indicates a lower ecological predictability than the hierarchically superordinated, specialized or functional movement skills (in overview Burton and Miller, 1998). In other words, not being able to shoot a free throw in general or all the more in a particular game situation might be a better explanation for physical inactivity-related social interaction problems and subsequent mental health problems than not being able to stand on one leg for a certain time for example. However, corresponding movement skills have an ontogenetic character and thus, respective findings are likely to suffer from a lack of cross-cultural validity. Moreover and particularly in the context of population-based, epidemiological research, functional and specialized movement skills are difficult to assess from a practical point of view since they require specific materials and/or ought to be performed in a specific movement context. One possible solution avoiding cross-cultural issues and combing ecological validity with aspects of practical feasibility would be the assessment of phylogenetic fundamental movement skills. Corresponding measures such as the Test of Gross Motor Development (TGMD; e.g., Wagner et al., 2016; Webster and Ulrich, in press) are also applicable to assess gross motor performances of children with DCD (Slater et al., 2010); when aiming to identify this particular group within a population-based sample, we propose to apply the full range of clinical-criteria including recommended motor assessments and by use of most restrictive cut-offs points. To that extent, Blank et al. (2014) recently published a German adaptation of the BOT which, together with the already existing German adaptation of the M-ABC (Petermann, 2011), should foster a more accurate DCD-diagnosis in German speaking countries and respective future studies.

Concerning a more detailed view on physical inactivity, information on duration, frequency, intensity and seasonality in different settings should be assessed. Corresponding data could be summarized to a minutes per week-based total physical activity score for example, whereby culture-specific settings would be ineffectual. Concerning the assessment of corresponding data, one has to keep in mind that self-reports are rather easy to administer in the context of epidemiological studies (e.g., Dishman et al., 2001) but also, that their validity suffers from certain under- or overestimations with increasing age, respectively (e.g., Prince et al., 2008). To overcome corresponding methodological constraints, self-reports on the amount of physical activity should be supplemented with objectively monitored (accelerometer-based; e.g., Cairney et al., 2015) data. To that extent and in terms of a meaningful estimation of total body-fat, BMI should be combined with more elaborated assessments such as the Bioelectrical Impedance Analysis (e.g., Cairney et al., 2005a).

When using the SDQ to screen for psychological distress as well as corresponding secondary risk and protective factors in future studies as recommended by Becker et al. (2015), we propose applying the most recent four-band categorization on the basis of multi-informant data (e.g., teachers; see also Goodman et al., 2000) which ought to be assessed by use of consistent methodology (e.g., written questionnaire).

Future elaborations of the Environmental Stress Hypothesis framework should primarily be focused on the postulated mediating and moderating effects. To that extent, our particular data suggests that peer-problems might be a comparatively weak operationalization of social interaction problems which opens the field for the assessment of different interaction partners such as parents or teachers (e.g., Missiuna et al., 2006). This all the more when considering that their supportive activities (e.g., Pless et al., 2001) are also interesting in terms of an extended assessment of social resources. Concerning personal resources and following recent cross-sectional research (e.g., Viholainen et al., 2014), an extended assessment should primarily be focused on measures of self-concept. Furthermore, additional measures of physical activity enjoyment (e.g., Jekauc et al., 2013b) promise an enhanced understanding of the relation between personal resources and physical inactivity (e.g., Cairney et al., 2007). Concerning mental health outcomes, a more comprehensive assessment of internalizing and externalizing problems is recommended. Finally, in accordance with Mancini et al. (2016), we opt for an age- and gender-specific elaboration of the Environmental Stress Hypothesis framework, whereby potential co-variates such as SES, ADHD or LD should be considered. However, respective interaction-analyses require the existence of substantial main effects. In matters of future gender-specific analysis our results indicate that girls are more likely to avoid organized physical activities as well as to bond with physically inactive peers, whereas boys are more likely to develop peer problems as well as an elevated body-mass.

Concerning practical implications our longitudinal data suggests that an elementary-school child with gross motor coordination problems is more likely to develop into an overweight adolescent who avoids organized physical activities, bonds with sedentary peers and shows either emotional or conduct problems. Thus, gross motor coordination problems (even when assessed on a basic skill level) apparently represent a serious issue for a healthy transition from childhood to adolescence which substantiates early movement interventions beyond the DCD population. Similarly to programs particularly designed for children with DCD (e.g., Missiuna et al., 2012), respective broader intervention strategies should be focused on improving children's participation in school and at home. Following our results, prerequisites for a successful integration seem good since only a small percentage of children with gross motor coordination problems show generalized peer problems or diminished prosocial behavior, respectively.

## Author contributions

MW: Substantially contributed to the conception and design of the work as well as to the analysis and interpretation of the data. Drafted the work and revised it critically for important intellectual content. Approved the version to be published. Is accountable for all aspects of the work in ensuring that questions related to the accuracy or integrity of any part of the work are appropriately investigated and resolved. DJ: Substantially contributed to the analysis and interpretation of the data. Revised the work critically for important intellectual content. Approved the version to be published. Is accountable for all aspects of the work in ensuring that questions related to the accuracy or integrity of any part of the work are appropriately investigated and resolved. ANW: Substantially contributed to the acquisition of the data. Revised the work critically for important intellectual content. Approved the version to be published. Is accountable for all aspects of the work in ensuring that questions related to the accuracy or integrity of any part of the work are appropriately investigated and resolved. AW: Substantially contributed to the acquisition of the data. Revised the work critically for important intellectual content. Approved the version to be published. Is accountable for all aspects of the work in ensuring that questions related to the accuracy or integrity of any part of the work are appropriately investigated and resolved.

## Funding

This work has been developed within the Motorik-Modul Longitudinal Study (MoMo) (2009–2021): Physical fitness and physical activity as determinants of health development in children and adolescents. MoMo is funded by the Federal Ministry of Education and Research (funding reference number: 01ER1503) within the research program 'long-term studies‘ in public health research.

### Conflict of interest statement

The authors declare that the research was conducted in the absence of any commercial or financial relationships that could be construed as a potential conflict of interest.
